# Antimicrobial activity of bacteriophage derived triple fusion protein against *Staphylococcus aureus*

**DOI:** 10.3934/microbiol.2019.2.158

**Published:** 2019-06-25

**Authors:** Natalia Y. Kovalskaya, Eleanor E. Herndon, Juli A. Foster-Frey, David M. Donovan, Rosemarie W. Hammond

**Affiliations:** 1 Floral and Nursery Plants Research Unit, U.S. National Arboretum, Agricultural Research Service, ORISE - U.S. Department of Agriculture, Beltsville, MD, USA; 2 Kerry's Nursery, Miami, FL, USA; 3 Animal Biosciences and Biotechnology Laboratory, U.S. Department of Agriculture, Agricultural Research Service, Beltsville, MD, USA; 4 Molecular Plant Pathology Laboratory, U.S. Department of Agriculture, Agricultural Research Service, Beltsville, MD, USA

**Keywords:** endolysins, lysostaphin, antimicrobials, CPMV-based vector, transient expression, codon optimization, *Nicotiana benthamiana*

## Abstract

The increasing spread of antibiotic-resistant microorganisms has led to the necessity of developing alternative antimicrobial treatments. The use of peptidoglycan hydrolases is a promising approach to combat bacterial infections. In our study, we constructed a 2 kb-triple-acting fusion gene (*TF*) encoding the N-terminal amidase-5 domain of streptococcal LambdaSA2 prophage endolysin (D-glutamine-L-lysin endopeptidase), a mid-protein amidase-2 domain derived from the staphylococcal phage 2638A endolysin (N-acetylmuramoyl-L-alanine amidase) and the mature version (246 residues) of the *Staphylococcus simulans* Lysostaphin bacteriocin (glycyl-glycine endopeptidase) at the C-terminus. The *TF* gene was expressed in *Nicotiana benthamiana* plants using the non-replicating *Cowpea mosaic virus* (CPMV)-based vector pEAQ-HT and the replicating *Alternanthera mosaic virus (*AltMV)-based pGD5TGB1_L88_23-MCS-CP3 vector, and in *Escherichia coli* using pET expression vectors pET26b+ and pET28a+. The resulting poor expression of this fusion protein in plants prompted the construction of a *TF* gene codon-optimized for expression in tobacco plants, resulting in an improved codon adaptation index (CAI) from 0.79 (*TF* gene) to 0.93 (*TFnt* gene). Incorporation of the *TF*nt gene into the pEAQ-HT vector, followed by transient expression in *N. benthamiana*, led to accumulation of TFnt to an approximate level of 0.12 mg/g of fresh leaf weight. Antimicrobial activity of purified plant- and bacterial-produced TFnt proteins was assessed against two strains of Gram-positive *Staphylococcus aureus* 305 and Newman. The results showed that plant-produced TFnt protein was preferentially active against *S. aureus* 305, showing 14% of growth inhibition, while the bacterial-produced TFnt revealed significant antimicrobial activity against both strains, showing 68 (IC_50_ 25 µg/ml) and 60% (IC_50_ 71 µg/ml) growth inhibition against *S. aureus* 305 and Newman, respectively. Although the combination of codon optimization and transient expression using the non-replicating pEAQ-HT expression vector facilitated production of the TFnt protein in plants, the most functionally active antimicrobial protein was obtained using the prokaryotic expression system.

## 1. Introduction

The increased use of antibiotics in human and animal healthcare has resulted in the emergence of antibiotic-resistant microorganisms. In this regard, *Staphylococcus aureus* is considered one of the most dangerous of all of the many common staphylococcal bacteria, having a high propensity to develop multi-drug resistance, and causing a number of serious diseases such as abscesses, respiratory infections, and food poisoning [1]–[3]. The development of resistance to antimicrobial drugs makes it difficult to find effective therapeutics. The most promising approaches to cope with antibiotic-resistant infection include the utilization of bacteriophage therapy [4]–[6] and the use of antimicrobial proteins such as peptidoglycan hydrolases (PGHs) produced by eukaryotic organisms (lysozyme) [7],[8], prokaryotic cells (exolysins/autolysins) [9],[10] and bacteriophages (endolysins/ectolysins) [11]–[14], that are responsible for degradation of peptidoglycan - the main structural component of the bacterial cell wall. PGHs of different origins, in combination with other PGHs or antibiotics, have been shown to demonstrate synergy [15]–[18]. The main advantages of bacteriophage therapy and phage lysins are their species specificity [19] that exclude the disturbance of indigenous microflora, their efficiency against Gram-positive [20]–[21] and Gram-negative [22]–[25] bacteria, and the fact of co-evolution of bacteriophages and their bacterial hosts makes resistance development unlikely [26],[27]. Thus, both bacteriophage therapy and phage lysins are prospective candidates as an alternative to broad range antibiotics in modern medicine, animal husbandry and agriculture.

In the present study, we constructed a triple-acting fusion (TF) gene consisting of enzymatically active domains of LambdsSA2 (λSA2) prophage endolysins, staphylococcal phage 2638A endolysins, and *Staphylococcus simulans* Lysostaphin bacteriocin. Similar TFs have been shown to be highly refractory to resistance development [28]. After codon optimization for expression in tobacco plants, the *TFnt* gene was transiently expressed in *Nicotiana benthamiana* leaves and then in *E. coli*. The antimicrobial properties of resulting proteins were examined against two strains *S. aureus* 305 and Newman *in vitro*.

## 2. Materials and methods

### 2.1. Microorganisms

The strains used include Gram-positive *Staphylococcus aureus* strain 305 (ATCC 29740), a gift from David Pritchard (University of Alabama, Birmingham, AL) and *Staphylococcus aureus* strain Newman (ATCC 25904), a gift from Jean Lee, Channing Lab, Brigham and Women's Hospital, Boston, MA). Staphylococcal bacteria were grown on Difco Tryptic Soy Agar (TSA) (BD, Sparks, MD, USA) and prior to antimicrobial assays the colonies were transferred into Bacto Tryptic Soy Broth (TSB) (BD) and incubated overnight at 37 °C with agitation at 230 rpm.

### 2.2. Plasmid constructions

#### 2.2.1. Cloning of the triple fusion gene into the plasmid vector pET21a+

The complete list of primers used for cloning is shown in Table 1. The triple fusion gene consisting of 1) a N-terminal amidase-5 domain (endopeptidase activity cleaves between D-glutamine and L-lysin) of LambdaSA2 prophage endolysin gene isolated from a Group B streptococcal genome [29]; 2) a mid-protein amidase-2 domain of staphylococcal phage 2638A endolysin gene (amidase activity cleaves between N-acetylmuramoyl-L-alanine) [30], and 3) a cDNA encoding the mature *Staphylococcus simulans* Lysostaphin (glycyl-glycine endopeptidase activity which cleaves the cross-linking pentaglycin bridges in the cell wall peptidoglycan of *S. aureus*) [31] at the C-terminus, was obtained by the following procedure.

**Table 1.** Oligonucleotide primers used for cloning.

Primer	Nucleotide sequence [5′→3′]^a^	Restriction sites
2438a146aaSalIF	ATGCATGCGTCGACGATGAAAAATCACAAGTATGTAG	*SalI*
2638a400aaXhoIR	AGTCAGTCCTCGAGAATGCCATCTTTATTCTG	*XhoI*
LysostaphinSalIF	AGCTAGCTGTCGACGCTGCAACACATGAACATTCAG	*SalI*
pET21aStyIR	CGTTTAGAGGCCCCAAGGGGTTATG	*StyI*
AgeTFF	TAACACCGGTATGGAAATCAACACTGAAATAGCCATTGC	*AgeI*
XhoTFR	TAATCTCGAGTTATCA*GTGATGGTGATGGTGATG*CTCTAGCTTTATAGTTCC	*XhoI*
AgeTFnt1F	TAACACCGGTATGGAGATAAATACTGAGATCGCAATCG	*AgeI*
XhoTFntR	TAATCTCGAGTTATCA*GTGATGGTGATGGTGATG*TTTGATTGTAC	*XhoI*
NcoTFnt F	TAAGCCATGGAGATAAATACTGAGATCGCAATCGC	*NcoI*
TFMluIF3	TAACACGCGTAACAATGGAAATCAACACTGAAATAGCCATTGC	*MluI*
TFSpeIR4	TAATACTAGTTCATTA*GTGATGGTGATGGTGATG*CTCGAGCTTTATAGTTCCCCACAGAACACC	*SpeI*

^a^Restriction sites are underlined; nucleotides in italics encode the 6xHis-tag.

A deletion construct, designated λSa2-E, was created from the pET21a/λSa2 (a gift from D. Pritchard [32]) as previously described [29]. The λSa2-E construct, which contains 435 bp encoding the endopeptidase domain of LambdaSA2 prophage endolysin gene, was digested with *Xho*I restriction enzyme for combining with the 2638A endolysin amidase-2 domain in the next step.

The amidase-2 domain (2638) of the staphylococcal phage 2638A endolysin gene (765 bp) was PCR amplified from the 2638A full-length construct using primer pair 2438a146aaSalIF/2638a400aaXhoIR and inserted into an *Xho*I-digested pET21a+/*λSa2-E*, giving rise pET21a+/*λSa2-E-2638*. Restriction enzymes *Sal*I and *Xho*I have compatible ends; when ligated in the correct orientation into the pET21a+/*λSa2-E*/XhoI, the *Xho*I site at the end of pET21a+/*λSa2-E* is destroyed, but the *Xho*I site at the end of 2638 gene is kept intact.

The mature Lysostaphin-encoding cDNA (738 bp) was PCR amplified using primer pair LysostaphinSalIF/pET21aStyIR and ligated into the *XhoI*-digested plasmid pET21a+/*λSa2-E*-*2638* (pET21a+/*λSa2-E*-*2638*/XhoI), giving rise pET21a+/*λSa2-E*-*2638*-*Lysostaphin* (pET21a+/*TF*). As in previous case, the ligation of *Sal*I and *Xho*I led to elimination of the *Xho*I site, but *Xho*I at the end of Lysostaphin gene remained intact. The final construct pET21a+/*TF* had a C-terminal 6xHis-tag for nickel column chromatography purification.

#### 2.2.2. Codon optimization

To facilitate triple fusion protein production in plants, the original *TF* gene sequence was optimized for expression in *Nicotiana tabacum* by Genscript (Piscataway, NJ, USA) and cloned into the pUC57 vector at the *Xba*I/*Sst*I restriction sites, giving rise pUC57/*TFnt*. The Codon Adaptation Index for *TF* and *TFnt* genes was calculated using the GenScript Rare Codon Analysis Tool (http://www.genscript.com/cgi-bin/tools/rare_codon_analysis).

#### 2.2.3. Cloning of the *TF* gene into an *Alternanthera mosaic virus* (AltMV)-based vector pGD5TGB1_L88_23-MCS-CP3

The coding region of the *TF* gene was amplified from the plasmid pET21a+/*TF* with primer pair TFMluIF3/TFSpeIR4 and, after standard cloning procedures, was incorporated into the pGD5TGB1_L88_23-MCS-CP3 vector [33]. The final construct pGD5TGB1_L88_23-MCS-CP3/*TF* contained a C-terminal 6xHis-tag to facilitate protein purification using Ni-NTA resin.

#### 2.2.4. Cloning of the *TF* and *TFnt* genes into a *Cowpea mosaic virus* (CPMV)-based vector pEAQ-HT

The coding regions of the *TF* and *TFnt* genes were amplified from the plasmids pET21a+/*TF* and pUC57/*TFnt* with two primer pairs AgeTFF/XhoTFR and AgeTFnt1F/XhoTFntR, respectively and, after standard cloning procedures, were incorporated into the CPMV-based pEAQ-HT vector [34]. The final constructs pEAQ-HT/*TF* and pEAQ-HT/*TFnt* contained a C-terminal 6xHis-tag to facilitate protein purification using Ni-NTA resin.

#### 2.2.5. Cloning of the *TFnt* gene into a pET26b+

The coding region of the *TFnt* gene was amplified from the plasmid pUC57/*TFnt* with primer pair NcoTFntF/XhoTFntR and, after standard cloning procedures, was incorporated into pET26b+ vector (Novagen, Madison, WI, USA). The final construct pET26b+/*TFnt* contained a C-terminal 6xHis-tag.

#### 2.2.6. Cloning of the *TFnt* gene into a pET28a+

The coding region of the *TFnt* gene was cloned into a plasmid vector pET28a+ at the *Nco*I/*XhoI* sites resulting in pET28a+/*TFnt*.

All constructs were verified by DNA sequencing.

### 2.3. Transformation of Agrobacterium tumefaciens and agroinfiltration

Introduction of pEAQ-HT/*TF*, pEAQ-HT/*TFnt* and pGD5TGB1_L88_23-MCS-CP3/*TF* constructs into *A. tumefaciens* strains LBA4404 and EHA105, respectively, and agroinfiltration of *N. benthamiana* leaves were performed as previously described [33],[35].

### 2.4. RT-PCR for detection of TF and TFnt genes in plants

Total cellular RNA was extracted by TRI Reagent (Molecular Research Center, Inc., Cincinnati, OH, USA) from infiltrated *N. benthamiana* leaves at 5 days post infiltration (dpi). RT-PCR analysis was carried out using the Titan One Tube RT-PCR System (Roche Molecular Biochemicals, Chicago, IL, USA) as described in the manufacturer's instructions with primer pair AgeTFnt1F/XhoTFntR (0.4 pmol, final concentration). For RT-PCR, 35 cycles were conducted in a GeneAmp®System 9700 (Applied Biosystems, Foster City, CA, USA) with AMV reverse transcriptase for the first strand cDNA synthesis and the Expand High Fidelity enzyme blend (Roche) consisting of Taq DNA polymerase and Tgo DNA polymerase for amplification of cDNA by PCR. The PCR fragments were fractionated on a 1.0% agarose gel.

### 2.5. Protein extraction from N. benthamiana and purification

Protein extraction from plant tissues was performed under denaturing conditions using the buffer included in the Ni-NTA Fast Start Kit (Qiagen, Valencia, CA, USA). After two rounds of centrifugation (at 4000 x g for 20 min and at 10000 x g for 10 min) followed by two steps of lysate filtration through 0.45 µm and 0.22 µm filters (Millipore Corporation, Bedford, MA, USA), the protein samples were purified using Ni-NTA Fast Start Kit (Qiagen) per the manufacturer's instructions. All buffers used for protein extraction and purification were supplied with 10% glycerol. Dialysis of the protein samples was performed with two buffer changes. The first buffer was 30 mM Tris HCl pH8.0 containing 10% glycerol and 0.1 mM DTT, followed by the second buffer – 30 mM Tris HCl pH8.0 containing 10% glycerol. The resulting protein samples were analyzed by Western blot analysis. Before performing antibacterial assays, the protein samples were concentrated using a centrifugal filter device YM-50 (Microcon, Millipore Corporation, Bedford, MA, USA) and filter sterilized with a Millex-GV 0.22 µm filter (Merck Millipore Ltd, Ireland). To determine the concentration of purified protein samples, measurement of absorbance at 280 nm was performed using a Thermo Scientific NanoDrop ND-8000 8-Sample Spectrophotometer.

### 2.6. Western blot analysis

An aliquot of the TFnt protein produced in plants was electrophoresed in a Novex® Tris-Glycine Gradient Gel (10 to 20%; Invitrogen, Gaithersburg, MD, USA) under denaturing conditions and then transferred to a nitrocellulose membrane according manufacturer's instructions (Invitrogen). The membrane was incubated with a 1:1000 dilution of polyclonal antibodies to Lysostaphin (AIC BIOTECH, Rockville MD, USA) followed by a 1:5000 dilution of goat anti-rabbit phosphatase-labeled antibodies (Kirkegaard & Perry Laboratories, Inc., Gaithersburg, MD, USA) with subsequent membrane development using the BCIP/NBT Membrane Phosphatase Substrate System (3-C) (Kirkegaard & Perry Laboratories, Inc).

### 2.7. Protein expression in E. coli

#### 2.7.1. Protein expression in *E. coli* at 37 °C and extraction

*E. coli* strain BL21 (DE3) (Stratagene, La Jolla, CA, USA) was used as a host for expression of the *TFnt* gene from pET26b+. The induction procedure for gene expression was as follows: 5 ml of Luria-Bertani broth (LB) containing kanamycin (50 µg/ml, final concentration) was inoculated with a bacterial colony and incubated overnight at 230 rpm at 37 °C. 0.5 ml of the overnight culture was transferred into a flask containing 50 ml of LB medium with the same antibiotic, and agitated at 230 rpm at 37 °C until the culture density reached an OD_600_ of 0.7–0.8 (Genesys 30 Visible Spectrophotometer, Thermo Scientific). IPTG (isopropyl-b-D-thiogalactopyranoside) was added to a final concentration of 0.5 mM with subsequent incubation at 37 °C for 3.5 hrs at 230 rpm. The bacterial cells were harvested by centrifugation at 4000 rpm for 20 min at 4 °C and stored at −20 °C. A non-induced culture was used as a negative control.

The BugBuster Master Mix Protein Extraction Reagent (Novagen) was used to extract the TFnt protein from bacterial cells. The extraction was carried out per manufacturer's instructions. The bacterial lysate was analyzed by SDS-PAGE followed by staining with SimplyBlue Safe Stain (Invitrogen). The protein fraction containing inclusion bodies (IBs) was used as a positive control for Western blot analysis of the plant-produced TFnt.

#### 2.7.2. Protein expression in *E. coli* at 10 °C, extraction and purification

*E. coli* strain BL21 (DE3) (Stratagene,) was used as a host for expression of the *TFnt* gene from pET26b+ and pET28a+ vectors. The induction procedure for gene expression was as follows: 5 ml of Luria-Bertani broth (LB) containing kanamycin (50 µg/ml, final concentration) was inoculated with a bacterial colony and incubated overnight at 230 rpm at 37 °C. 0.5 ml of overnight culture was transferred into a flask containing 50 ml of LB medium with the same antibiotic, and agitated at 230 rpm at 37 °C until the culture density reached an OD_600_ of 0.5–0.6 (Genesys 30 Visible Spectrophotometer, Thermo Scientific). IPTG was added to final concentration 1.5 mM with subsequent incubation at 10 °C for 20 hrs at 190 rpm. The bacterial cells were harvested by centrifugation at 4000 rpm for 20 min at 4 °C and stored at −20 °C. A non-induced culture was used as a negative control.

The BugBuster Master Mix Protein Extraction Reagent (Novagen) was used to extract the TFnt protein from bacterial cells. The extraction was carried out per manufacturer's instructions. The bacterial lysate was analyzed by SDS-PAGE followed by staining with SimplyBlue Safe Stain (Invitrogen). The soluble TFnt protein was purified with Ni-NTA His·Bind Resin (EMD Millipore Corp., USA) under native conditions using the Ni-NTA Buffer Kit (Novagen) per manufacturer's instructions. All buffers used for protein purification were supplied with glycerol (30% final concentration) including protein sample that was added to the Ni-NTA His·Bind Resin. The resulting protein was analyzed with SDS-PAGE. Before performing antibacterial assays, the protein samples were concentrated with the Amicon Ultra-0.5 centrifugal filter device Ultracel-10K (Merck Millipore Ltd.) and filter sterilized with a Millex-GV 0.45 µm filter (Merck Millipore Ltd.). The protein concentration was measured using the Quick Start Bradford 1x Dye Reagent (Bio-Rad Laboratories, Hercules, CA, USA) and Quick Start Bovine Serum Albumin (BSA) Standard Set (Bio-Rad).

### 2.8. Antibacterial assay

#### 2.8.1. Growth inhibition assay

The antibacterial activity of the purified bacterial- and plant-produced TFnt protein was examined against *S. aureus* 305 and Newman according to the procedures previously described [36] using a final protein concentrations 50 µg/ml (bacterial-produced protein) and 100 µg/ml (bacterial- and plant-produced protein). The bacterial concentration at the onset of the experiment was 1.5 × 10^8^ colony-forming units (CFU) per ml for *S. aureus* 305, and 3 × 10^8^ CFU/ml for *S. aureus* Newman. The reaction mixtures were incubated at 37 °C with shaking at 230 rpm for 3.5 hrs for each culture. Following incubation, 50 µl aliquots of protein-treated bacterial cultures were serially diluted in sterile water (from 10^−2^ to 10^−6^) and 20 µl of diluted bacterial suspensions were plated onto tryptic soy agar (TSA, Difco Sparks, MD) media. The plates were incubated at 37 °C overnight and the bacterial growth was examined by counting CFU. Each experiment had at least three repetitions with triplicate platings of each sample. The data are expressed as the mean ± the standard error of the mean. Statistical significance of obtained data was analyzed by calculating the p-value (http://www.socscistatistics.com).

#### 2.8.2. Plate lysis assay

Two strains *S. aureus* 305 and Newman were used as a test cultures in a plate lysis assay. Eight ml of tryptic soy broth (TSB, Difco) was inoculated with 0.4 ml of overnight cultures of *S. aureus* 305 and Newman. The tubes were incubated at 37 °C at 230 rpm until they reached an OD_600_ of 0.4–0.6. Two ml of culture was pipetted on square gridded plates with TSA and spread evenly by tilting the plates. Excess culture was removed by pipet and the plates were left to dry for about 20 min under the laminar flow hood. Ten µl aliquots of the TFnt protein at 0.7 mg/ml was pipetted on the gridded plates containing the test bacteria and left to dry for about 20 min. The plates were then kept at 37 °C overnight. The results were captured with a FluorChem SP Gel Imaging System (Alpha Innotech, San Leandro, CA).

## 3. Results

**Figure 1.**
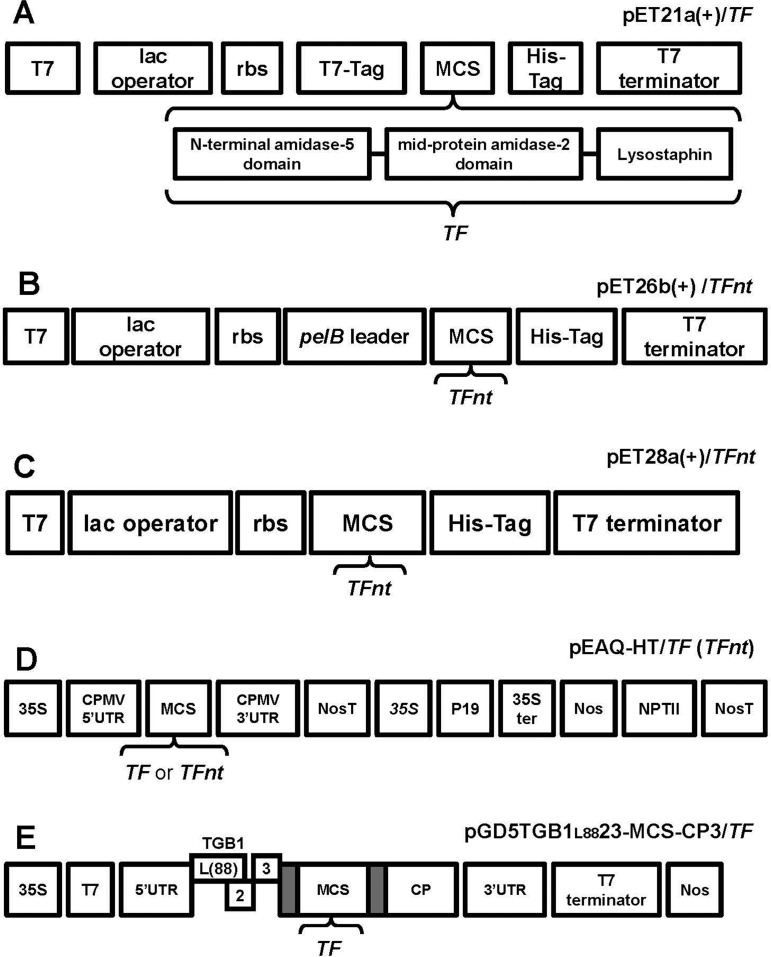
Schematic representation of vector constructions carrying *TF* and *TFnt* genes with major features labeled. **A**: The cloning/expression region of pET21a+/*TF* vector indicating the insertion of the *TF* gene consisting of N-terminal amidase-5 domain (D-glutamine-L-lysin endopeptidase activity) of LambdaSA2 prophage endolysin, a mid-protein amidase-2 domain of staphylococcal phage 2638A endolysin (amidase activity) and the mature *S. simulans* Lysostaphin (glycyl-glycine endopeptidase activity). pET21a+ carries an N-terminal T7-Tag sequence plus optional C-terminal His-Tag sequence. rbs—ribosomal binding site sequence; MCS—multiple cloning site. **B**: The cloning/expression region of pET26b+/*TFnt* vector indicating the insertion of the *TFnt* gene. pET26b+ carries an N-terminal *pelB* signal sequence for potential periplasmic localization of target gene. **C**: The cloning/expression region of pET28a+/*TFnt* vector indicating the insertion of the *TFnt* gene. **D**: The cloning/expression region of pEAQ-HT vector indicating the insertion of the *TF* and *TFnt* genes. 35S—*Cauliflower mosaic virus* 35S (35S) promoters; NosT—nopaline synthase transcriptional terminator; Nos—nopaline synthase promoter sequence; P19—gene coding the suppressor of gene silencing; NPTII—kanamycin resistance gene. **E**: The cloning/expression region of bipartite vector pGD5TGB1_L88_23-MCS-CP3 indicating the insertion of the *TF* gene. TGB1 (L(88))—‘triple gene block 1’ with Leucine at the position 88 (Lim et al., 2010); gray boxes - duplicated CP (coat protein) subgenomic promoters.

The *TF* gene encoding three unique PGHs including an N-terminal amidase-5 domain (D-glutamine-L-lysin endopeptidase activity) of LambdaSA2 prophage endolysin, a mid-protein amidase-2 domain of staphylococcal phage 2638A endolysin (amidase activity) and the mature *S. simulans* Lysostaphin (glycyl-glycine endopeptidase activity) was sub-cloned into the pET21a+ plasmid vector (Figure 1A). To produce the TF protein in plants, the *TF* gene was incorporated into CPMV-based vector pEAQ-HT (non-replicating system) (Figure 1D) and the AltMV-based vector pGD5TGB1_L88_23-MCS-CP3 (replicating system) (Figure 1E) with subsequent introduction into *N. benthamiana* plants through agroinfiltration. The infiltrated plants did not exhibit any symptoms and were indistinguishable from control, non-infiltrated plants. RT-PCR analysis of total RNA extracted on 5 dpi revealed the presence of *TF* gene in leaves infiltrated with pEAQ-HT/*TF*, whereas in the case of infiltration with pGD5TGB1_L88_23-MCS-CP3/*TF*, no *TF* gene was detected (data not shown). Western blot analysis did not reveal TF protein production in both cases (data not shown).

**Figure 2.**
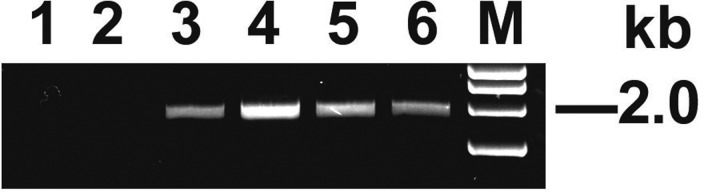
RT-PCR analysis of total cellular RNA isolated from agroinfiltrated *N. benthamiana* leaves (5 dpi). Lanes: **1**: ‘control-mock’ (healthy plant); **2**: ‘empty-pEAQ-HT’; **3–6**: ‘pEAQ-HT/*TFnt*’; ‘M’: DNA marker: Hyper Ladder 1kb (Bioline Taunton, MA).

To overcome the inability to produce the TF protein in plants, the *TF* gene sequence was codon-optimized for expression in tobacco plants, giving rise to *TFnt*, and incorporated into the pEAQ-HT vector under control of the *Cauliflower mosaic virus* 35S promoter, with subsequent introduction into *N. benthamiana* through agroinfiltration. The plant codon-optimized sequence of *TFnt* is presented in Supplementary materials. After codon optimization, the codon adaptation index (CAI) increased from 0.79 for the *TF* gene to 0.93 for the optimized *TFnt* gene sequence. The expression of *TFnt* gene resulted in distinct yellowing of infiltrated leaves on 5 dpi, in some cases accompanied by appearance of lesion zones around the infiltrated area on 7 dpi, with subsequent leaf collapse on 8–9 dpi. To avoid leaf collapse, the bacterial concentration of the *A. tumefaciens* culture used for infiltration was decreased from OD_600_ = 0.6 to 0.2; however, this led to more rapid tissue necrosis on 6 dpi. The presence of the *TFnt* gene in infiltrated leaves was analyzed on 4 dpi, in order to collect plant material for protein extraction before leaf collapse occurred, but in this case, the target gene was detected in less than 4% of infiltrated leaves. Thus, in our experiments, we used a concentration OD_600_ = 0.6 of the *A. tumefaciens* culture for plant infiltration. The stability of *TFnt* transcript resulting from *in vivo* transcription of the insertion within the plasmid vector was demonstrated by RT-PCR assays performed on RNA samples isolated from infiltrated leaves on 5 dpi (Figure 2).

**Figure 3.**
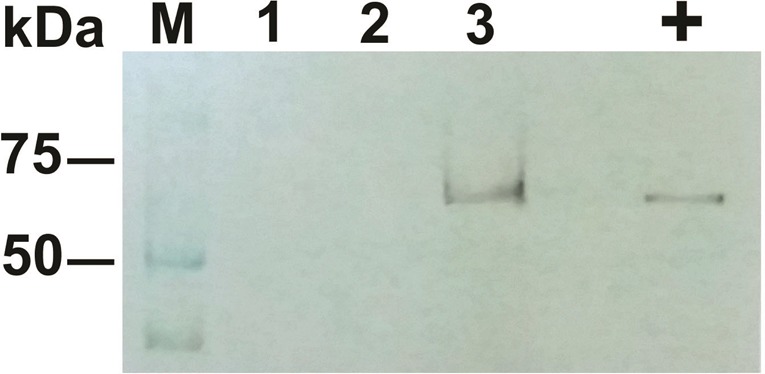
Western blot analysis of TFnt produced in *N. benthamiana* on 6 dpi. The membrane was incubated with a 1:1000 dilution of polyclonal antibodies to Lysostaphin (AIC BIOTECH, Rockville MD, USA) followed by a 1:5000 dilution of goat anti-rabbit phosphatase-labeled antibodies (Kirkegaard & Perry Laboratories (KPL), Inc., Gaithersburg, MD) with subsequent membrane development by BCIP/NBT Membrane Phosphatase Substrate System (KPL). Lanes: **M:** Precision Plus Protein Kaleidoscope (Bio-Rad); **1:** ‘control-mock’ (healthy plant); **2:** ‘empty-pEAQ-HT’; **3:** ‘pEAQ-HT/*TFnt*’; **+** - positive control (*E. coli* produced TFnt). The predicted TFnt MW = 72.9 kDa.

Although SDS-PAGE Simply Blue Safe Staining did not show the presence of the Ni-NTA-purified TFnt due to low protein concentration (data not shown), Western blot analysis using Lysostaphin-specific antibodies confirmed TFnt protein production in plants on 6 dpi (Figure 3).

**Figure 4.**
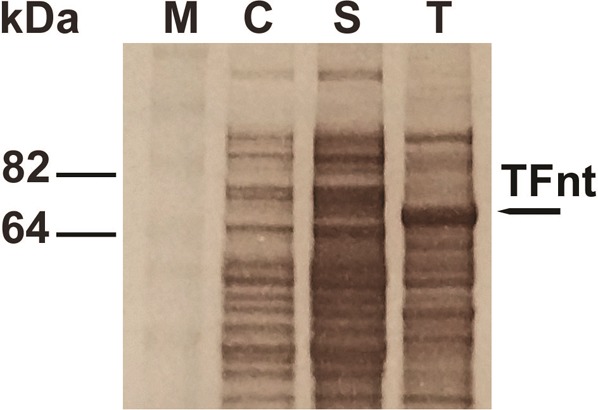
SDS-PAGE analysis of protein fractions produced in *E. coli* BL21 (DE3) transformed with pET26b+/*TFnt*. Lanes: **C**: uninduced control (without addition of IPTG), **S -** soluble protein fraction; **T**: total protein fraction that contains TFnt localized in inclusion bodies. The latter fraction (**T**) has been used as positive control for Western blot analysis of TFnt protein. kDa = BenchMark Pre-Stained Protein Ladder (Novex, Life Technologies, CA, USA). The predicted TFnt MW = 72.9 kDa.

The accumulation of TFnt protein in *N. benthamiana* was estimated at 0.12 mg/g of fresh leaf weight. Although the *TFnt* gene was optimized for expression in plants, the modified protein was also expressed in *E. coli* BL21 (DE3) using pET26b+ (Figure 1B) and pET28a+ (Figure 1C) vectors. When using pET26b+ vector and protein induction at 37 °C, SDS-PAGE analysis of *E. coli* lysate revealed localization of TFnt in inclusion bodies (Figure 4). This protein sample was utilized as a positive control for Western blot assays.

**Figure 5.**
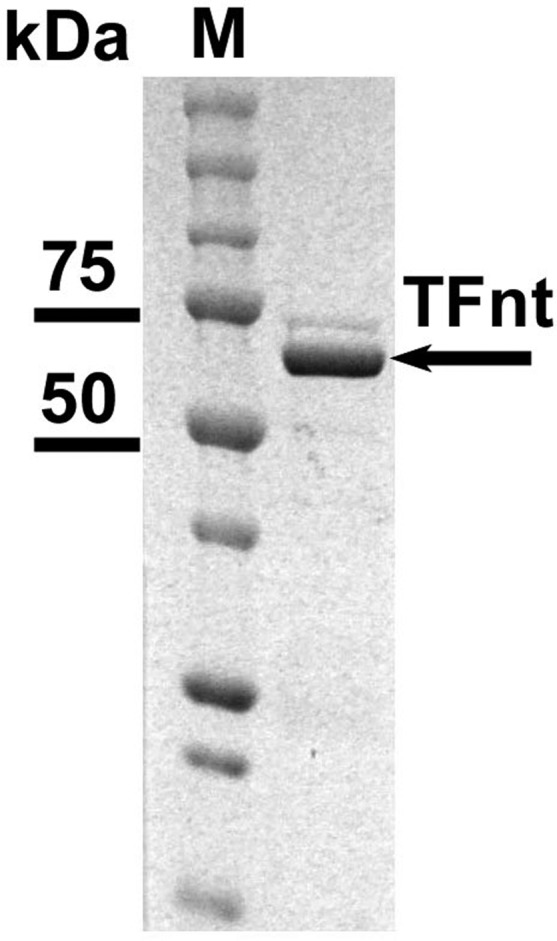
SDS-PAGE analysis of Ni-NTA purified TFnt protein produced in *E. coli* BL21 (DE3) transformed with pET28a+/*TFnt*. **M**: Precision Plus Protein All Blue Prestained Protein Standards (Bio-Rad). The predicted TFnt MW = 72.9 kDa.

The expression of the *TFnt* gene using pET26b+ and pET28a+ vectors at 10 °C led to soluble protein production (see Materials and methods). In our experiments, the pET28a+ vector resulted in better gene expression and protein production at the low temperature than the pET26b+ vector (data is not shown). The resulting soluble TFnt protein was purified (Figure 5), filter sterilized and used in the antimicrobial assays.

**Figure 6.**
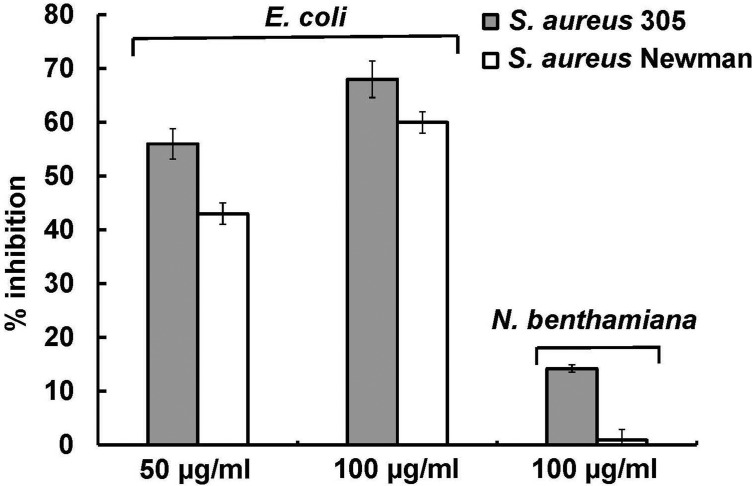
Antimicrobial activity of bacterial- and plant-produced TFnt protein against *S. aureus* 305 and Newman in a growth inhibition assay. % inhibition on the Y-axis refers to growth inhibition of *S. aureus* 305 and Newman. The TFnt concentration is indicated below the X-axis. Inhibition of *S. aureus* was statistically significant (p < 0.05) for all experimental groups except for inhibition of *S. aureus* Newman by plant-produced TFnt.

To examine the antimicrobial activity of purified plant-produced TFnt against *S. aureus* 305 and Newman, the growth inhibition assay with counting bacterial CFUs was performed as described in Materials and methods. The results revealed that only *S. aureus* 305 was susceptible to TFnt treatment, showing a statistically significant 14% of growth inhibition (Figure 6).

**Figure 7.**
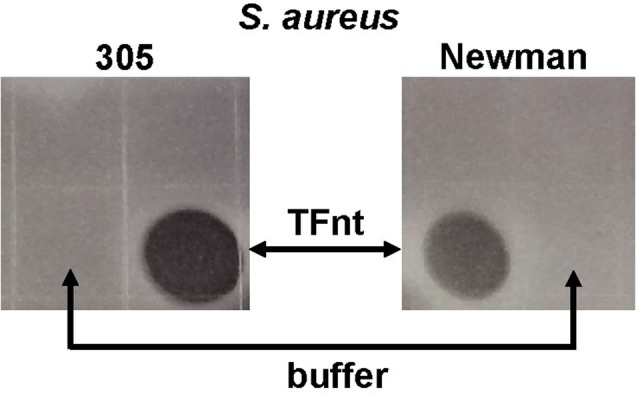
Antimicrobial activity of bacterial produced TFnt protein (7 µg) against *S. aureus* 305 and Newman in plate lysis assay. *Staphylococcus* strains were applied to the plate as described in Materials and methods. Ten µl spots of samples were applied to the agar plate and the plate was incubated at room temperature. A photographic image of the plate was taken 16 hrs after application of the samples.

The antimicrobial activity of bacterial-produced TFnt was examined using the growth inhibition and plate lysis assays. The growth inhibition assay revealed significant antibacterial activity of TFnt against both staphylococcal strains (Figure 6). It was shown that bacterial-produced TFnt inhibited *S. aureus* 305 and Newman by 68 and 60%, respectively, at a final protein concentration of 100 µg/ml. The plate lysis assay also revealed the pronounced antimicrobial activity of TFnt protein (7 µg) expressed as discrete cleared spots on a lawn of both bacterial strains (Figure 7).

## 4. Discussion

The search for new antimicrobials that can substitute for antibiotics and combat the drug-resistant pathogens has becomes a priority in medicine, veterinary care and food safety nationwide.

In the present study, we engineered a TF protein consisting of three protein domains possessing different lytic activities: N-terminal amidase-5 domain (D-glutamine - L-lysin endopeptidase) of LambdaSA2 prophage endolysin, a mid-protein amidase-2 domain (N-acetylmuramoyl - L-alanine amidase) of staphylococcal phage 2638A endolysin and the mature *S. simulans* Lysostaphin (glycyl-glycine endopeptidase activity). LambdaSA2 prophage endolysin, staphylococcal phage 2638A endolysin and Lysostaphin are known to be active against number of Gram-positive streptococci and staphylococci [28]–[30].

To produce the TF protein in plants, non-replicating (CPMV-based pEAQ-HT vector) and replicating (AltMV-based pGD5TGB1_L88_23-MCS-CP3 vector) expression systems were employed. Although no TF protein was detected for both expression systems, RT-PCR analysis showed the stability of *TF* gene only within pEAQ-HT vector. Most likely, the instability of *TF* gene in replicating pGD5TGB1_L88_23-MCS-CP3 vector was related to the size of inserted gene (2 kb). It has been reported that a disadvantage of expression vectors based on replicating RNA viruses is the limited size of the heterologous gene that can be expressed and the genetic instability of the construct during replication of the viral genome [34],[37],[38]. This observation is in agreement with our previous unsuccessful attempts to express a 1.9 kb-gene consisting of the staphylococcal phage K endolysin (LysK), and Lysostaphin within replicating *Potato virus* X- based vector (unpublished data).

To overcome the problem with expression of the TF protein in plants, the *TF* gene was codon-optimized for expression in tobacco plants and the modified *TFnt* gene was incorporated into the pEAQ-HT vector and introduced in *N. benthamiana*. Codon optimization often improves protein production in living organisms by increasing the translational efficiency of the target gene by transforming the DNA nucleotide sequence of one species into DNA nucleotide sequence of another species [39]–[42].

Expression of the *TFnt* gene in plants resulted in distinct yellowing of infiltrated leaves with subsequent leaf collapse. It has been reported that high concentrations of infiltrated *Agrobacterium* cultures can trigger a hypersensitive response in the infiltrated tissues [43]. Chen et al., 2013 [44] demonstrated that OD_600_ = 0.12 is the optimal concentration for *A. tumefaciens* that provides maximum transgene delivery without tissue damage. In our experiments, decreasing the *A. tumefaciens* concentration from OD_600_ = 0.6 to 0.2 before infiltration led to even more rapid tissue collapse. Perhaps, this unexpected reaction of plant tissues was caused by toxicity of the TFnt protein rather than by the infiltrated culture. We hypothesize that the use of a low concentration of *Agrobacterium* cells for infiltration may have maximized transgene delivery, decreased the influence of the infiltrated culture and therefore increased gene expression causing more rapid tissue necrosis. The low level of TFnt protein production in plants may be related to the toxicity of the TFnt protein for plant tissues. In previous studies, we have observed the toxicity of bacteriophage endolysin proteins when expressed in plants [45].

Because of poor expression of the fusion protein in *N. benthamiana*, the TFnt was produced in prokaryotic expression system. To do so, the pET26b+ vector containing an N-terminal pelB signal sequence fused to the expression protein for facilitating protein localization to the periplasm and eliminate IB formation was employed. However, SDS-PAGE analysis revealed that the 72.9 kDa-target protein was localized in IBs when expressed at 37 °C. Among other reasons, the probability of expressing a soluble protein decreases considerably at molecular weights above 60 kDa [46]. Decreasing the temperature during induction until 10 °C and using the pET28a+ vector for gene expression resulted in successful TFnt protein production in *E. coli*. It was reported that lowering of the temperature during induction (10–25 °C) facilitates obtaining soluble protein, and may be due to slower rates of protein production that allows newly transcribed recombinant proteins sufficient time to fold properly [30],[46],[47].

The antimicrobial assays showed that the most functionally active TFnt protein was produced in prokaryotic expression system, revealing significant growth inhibition and lysis of both staphylococcal strains when compared to the activity of plant-produced protein. Despite numerous advantages of plants as a host for gene expression [48],[49], the low level of protein production remains the main drawback of this system [48].

In conclusion, although the combination of codon optimization and transient expression using the non-replicating pEAQ-HT expression vector facilitated production of TFnt protein in plants, the most functionally active antimicrobial protein was obtained using the prokaryotic expression system, the advantages of which are described [50]. According to the results of our research, significant antimicrobial activity of TFnt protein makes it a promising candidate for the further evaluation against infections caused by staphylococcal bacteria *in vivo*. The long-term goal of our project is to obtain plants producing antimicrobials that could be used as a supplement for the forage in animal husbandry to control or treat staphylococcal infections.
