# Supplementary material for: Antimicrobial activity of bacteriophage derived triple fusion protein against Staphylococcus aureus
Source: AIMS Microbiol. 2019 Jun 25;5(2):158–75. doi: 10.3934/microbiol.2019.2.158 (PMC6642909; doi:10.3934/microbiol.2019.2.158)
Supplement: Supplementary file 1 [file microbiol-05-02-158-s001.pdf]

AIMS Microbiology

http://www.aimspress.com/journal/microbiology

AIMS Microbiology, 5(2): 158–175.
DOI: 10.3934/microbiol.2019.2.158
Received: 18 March 2019
Accepted: 19 June 2019
Published: 25 June 2019

Research article

Antimicrobial activity of bacteriophage derived triple fusion protein against *Staphylococcus aureus*

Natalia Y. Kovalskaya[1*], Eleanor E. Herndon[2], Juli A. Foster-Frey[3], David M. Donovan[3] and Rosemarie W. Hammond[4]

[1] Floral and Nursery Plants Research Unit, U.S. National Arboretum, Agricultural Research Service, ORISE - U.S. Department of Agriculture, Beltsville, MD, USA
[2] Kerry's Nursery, Miami, FL, USA
[3] Animal Biosciences and Biotechnology Laboratory, U.S. Department of Agriculture, Agricultural Research Service, Beltsville, MD, USA
[4] Molecular Plant Pathology Laboratory, U.S. Department of Agriculture, Agricultural Research Service, Beltsville, MD, USA

* **Correspondence:** Email: Natalia.Kovalskaya@ars.usda.gov; Phone: 3015045203; FAX: 3015045449.

```
1     ATGGAAATCAACACTGAAATAGCCATTGCCTGGATGTCTGCACGTCAAGGCAA     TF
      ATGGAGATAAATACTGAGATCGCAATCGCATGGATGTCCGCAAGGCAGGGAAA     TFnt

54    GGTCAGCTATTCCATGGACTACCGTGACGGCCCTAACAGCTATGACTGTTCCA     TF
      GGTGTCCTACTCTATGGATTACAGGGATGGTCCTAACTCATACGATTGTTCTT     TFnt

107   GCTCTGTCTATTATGCTCTCAGGTCAGCTGGTGCAAGCTCAGCAGGTTGGGCG     TF
      CATCTGTTTACTACGCTCTTAGATCTGCTGGAGCTTCATCTGCTGGTTGGGCT     TFnt

160   GTCAACACTGAGTACATGCACGATTGGCTGATTAAAAACGGTTATGAGCTTAT     TF
      GTTAATACTGAATACATGCATGATTGGCTTATTAAGAATGGTTACGAATTGAT     TFnt

213   TGCTGAAAACGTGGATTGGAATGCTGTCCGTGGTGATATAGCAATTTGGGGCA     TF
      CGCTGAGAATGTTGATTGGAATGCTGTTAGAGGAGATATTGCTATTTGGGGAA     TFnt
```

266	TGCGAGGGCACTCAAGCGGAGCTGGTGGTCATGTAGTCATGTTTATTGACCCT	TF
	TGAGGGGTCATTCATCTGGTGCTGGAGGTCATGTTGTTATGTTCATCGATCCA	TFnt
319	GAAAATATCATTCACTGTAACTGGGCAAATAATGGCATCACAGTGAACAACTA	TF
	GAGAACATCATCCATTGCAACTGGGCTAATAACGGAATCACTGTTAATAACTA	TFnt
372	CAATCAGACAGCGGCTGCTAGTGGCTGGATGTATTGCTACGTTTACCGTTTGA	TF
	CAATCAAACAGCTGCTGCTTCAGGTTGGATGTATTGTTACGTTTATAGGCTTA	TFnt
425	AAAGCGGAGCTCTCGACGATGAAAAATCACAAGTATGTAGTGGTTTGGCTATG	TF
	AGTCTGGAGCTTTGGATGATGAAAAATCACAAGTTTGCTCTGGTCTTGCTATG	TFnt
478	GAAAAATATGACATTACAAATTTAAATGCTAAACAAGATAAATCAAAGAATGG	TF
	GAGAAGTACGATATTACTAATTTGAACGCTAAGCAGGATAAGTCTAAGAATGG	TFnt
531	GAGCGTGAAAGAGTTGAAACATATCTATTCAAACCATATTAAAGGTAACAAGA	TF
	TTCTGTTAAGGAACTTAAGCATATCTATTCAAACCATATTAAGGGTAATAAGA	TFnt
584	TTACAGCACCAAAACCTAGTATTCAAGGTGTGGTCATCCACAATGATTATGGT	TF
	TTACAGCTCCAAAACCTTCTATCCAAGGAGTTGTTATTCATAACGATTACGGT	TFnt
637	AGTATGACACCTAGTCAATACTTACCATGGTTATATGCACGTGAGAATAACGG	TF
	TCAATGACTCCATCTCAATACCTTCCTTGGTTGTATGCTAGAGAGAATAACGG	TFnt
690	TACACACGTTAACGGTTGGGCTAGTGTTTATGCAAATAGAAACGAAGTGCTTT	TF
	AACACATGTTAATGGTTGGGCTTCTGTTTATGCTAATAGGAATGAGGTTCTTT	TFnt
743	GGTATCATCCGACAGACTACGTAGAGTGGCATTGTGGTAATCAATGGGCAAAT	TF
	GGTACCATCCAACTGATTATGTTGAGTGGCATTGTGGAAATCAATGGGCTAAC	TFnt
796	GCTAACTTAATCGGATTTGAAGTGTGTGAGTCGTATCCTGGTAGAATCTCGGA	TF
	GCTAATTTGATCGGTTTCGAAGTTTGCGAGTCATACCCTGGAAGAATTTCTGA	TFnt
849	CAAATTATTCTTAGAAAATGAAGAAGCGACATTGAAAGTAGCTGCGGATGTGA	TF
	TAAGCTTTTTCTTGAAAATGAGGAGGCTACATTGAAGGTTGCTGCTGATGTTA	TFnt
902	TGAAGTCGTACGGATTACCAGTTAATCGCAACACTGTACGTCTGCATAACGAA	TF
	TGAAATCATACGGACTTCCAGTTAACAGAAACACTGTTAGGTTGCATAATGAG	TFnt
955	TTCTTCGGAACTTCTTGTCCACATCGTTCGTGGGACTTGCATGTTGGCAAAGG	TF
	TTTTTCGGTACATCATGTCCACATAGATCTTGGGATCTTCATGTTGGTAAAGG	TFnt
1008	TGAGCCTTACACAACTACTAATATTAATAAAATGAAAGACTACTTCATCAAAC	TF

```
     AGAGCCTTACACTACAACTAATATTAATAAGATGAAAGATTACTTTATTAAGA     TFnt

1061 GCATCAAACATTATTATGACGGTGGAAAGCTAGAAGTAAGCAAAGCAGCAACT     TF
     GGATTAAACATTATTACGATGGAGGAAAGCTTGAAGTTTCTAAAGCTGCTACT     TFnt

1114 ATCAAACAATCTGACGTTAAGCAAGAAGTTAAAAAGCAAGAAGCAAAACAAAT     TF
     ATTAAGCAATCTGATGTTAAACAAGAAGTTAAGAAACAAGAGGCTAAGCAAAT     TFnt

1167 TGTGAAAGCAACAGATTGGAAACAGAATAAAGATGGCATTCTCGACGCTGCAA     TF
     TGTTAAAGCTACTGATTGGAAGCAAAATAAGGATGGTATTCTTGATGCTGCTA     TFnt

1220 CACATGAACATTCAGCACAATGGTTGAATAATTACAAAAAAGGATATGGTTAC     TF
     CACATGAGCATTCTGCTCAATGGTTGAATAACTACAAGAAAGGTTACGGATAT     TFnt

1273 GGCCCTTATCCATTAGGTATAAATGGCGGTATGCACTACGGAGTTGATTTTTT     TF
     GGTCCATATCCTCTTGGAATTAATGGTGGAATGCATTACGGTGTTGATTTCTT     TFnt

1326 TATGAATATTGGAACACCAGTAAAAGCTATTTCAAGCGGAAAAATAGTTGAAG     TF
     TATGAACATCGGAACACCTGTTAAGGCTATTTCATCTGGAAAAATTGTTGAAG     TFnt

1379 CTGGTTGGAGTAATTACGGAGGAGGTAATCAAATAGGTCTTATTGAAAATGAT     TF
     CTGGTTGGTCAAATTATGGAGGTGGAAATCAAATTGGACTTATTGAGAATGAT     TFnt

1432 GGAGTGCATAGACAATGGTATATGCATCTAAGTAAATATAATGTTAAAGTAGG     TF
     GGTGTTCATAGACAATGGTACATGCATTTGTCTAAGTATAACGTTAAAGTTGG     TFnt

1485 AGATTATGTCAAAGCTGGTCAAATAATCGGTTGGTCTGGAAGCACTGGTTATT     TF
     AGATTACGTTAAGGCTGGTCAAATTATTGGATGGTCAGGTTCTACTGGATATT     TFnt

1538 CTACAGCACCACATTTACACTTCCAAAGAATGGTTAATTCATTTTCAAATTCA     TF
     CAACAGCTCCACATCTTCATTTCCAAAGGATGGTTAACTCATTTTCTAACTCA     TFnt

1591 ACTGCCCAAGATCCAATGCCTTTCTTAAAGAGCGCAGGATATGGAAAAGCAGG     TF
     ACTGCTCAAGATCCAATGCCATTTCTTAAGTCTGCTGGATATGGTAAAGCTGG     TFnt

1644 TGGTACAGTAACTCCAACGCCGAATACAGGTTGGAAAACAAACAAATATGGCA     TF
     TGGAACAGTTACTCCAACACCTAATACTGGATGGAAGACAAATAAGTACGGTA     TFnt

1697 CACTATATAAATCAGAGTCAGCTAGCTTCACACCTAATACAGATATAATAACA     TF
     CTCTTTATAAGTCTGAATCAGCTTCTTTTACTCCTAACACAGATATTATCACA     TFnt

1750 AGAACGACTGGTCCATTTAGAAGCATGCCGCAGTCAGGAGTCTTAAAAGCAGG     TF
     AGAACAACTGGACCTTTTAGATCAATGCCTCAATCTGGAGTTTTGAAGGCTGG     TFnt
```

1803 TCAAACAATTCATTATGATGAAGTGATGAAACAAGACGGTCATGTTTGGGTAG TF
TCAAACTATCCATTACGATGAGGTTATGAAACAAGATGGTCATGTTTGGGTTG TFnt

1856 GTTATACAGGTAACAGTGGCCAACGTATTTACTTGCCTGTAAGAACATGGAAT TF
GTTACACAGGAAATTCTGGTCAAAGGATATATCTTCCTGTTAGGACTTGGAAC TFnt

1909 AAGTCTACTAATACTCTGGGTGTTCTGTGGGGAACTATAAAGCTAGAG*CATCA* TF
AAATCTACAAATACATTAGGAGTTCTCTGGGGTACAATCAAAXXXXXX*CATCA* TFnt

1962 *CCATCACCATCAC*TGA TF
*CCATCACCATCAC*TGA TFnt

Figure S1. Plant-codon opti üzed sequence of *TF* gene. Upper line is sequence of original *TF* gene and lower line is codon-opti üzed sequence of *TF* gene *(TFnt)*. Yellow boxes indicate modified sequences. Green box shows mutated restriction site that was present in original *TF* gene sequence and is absent in codon-opti üzed *TFnt* gene. Underline: start (ATG) and stop (TGA) codons. Italics: 6x His-tag

AIMS Press

© 2019 the Author(s), licensee AIMS Press. This is an open access article distributed under the terms of the Creative Commons Attribution License (http://creativecommons.org/licenses/by/4.0)
